# Women’s suggestions on how to improve the quality of maternal and newborn care: A qualitative analysis from the IMAgiNE EURO survey in Italy during the two years of the COVID-19 pandemic

**DOI:** 10.18332/ejm/192143

**Published:** 2024-10-22

**Authors:** Simona Fumagalli, Antonella Nespoli, Laura Iannuzzi, Ilaria Mariani, Emanuelle Pessa Valente, Marzia Lazzerini

**Affiliations:** 1 School of Medicine and Surgery, University of Milano-Bicocca, Monza, Italy; 2 Department of Obstetrics, Foundation IRCCS San Gerardo dei Tintori, Monza, Italy; 3 Centre for Midwifery, Maternal and Perinatal Health, Bournemouth University, Bournemouth, United Kingdom; 4 WHO Collaborating Centre for Maternal and Child Health, Institute for Maternal and Child Health IRCCS Burlo Garofolo, Trieste, Italy

**Keywords:** women, quality of care, qualitative research, COVID-19, childbirth, WHO standards

## Abstract

**INTRODUCTION:**

Collecting women’s views and suggestions for improving quality of maternal–newborn care (QMNC) is a crucial aspect of maternity care evaluation often overlooked in Italy and globally. Childbearing women experienced numerous challenges during the COVID-19 pandemic including the rapid and significant reorganization of maternity services and care. Their perspective on what to prioritize for QMNC improvement is hence pivotal. The aim of this study was to explore maternal suggestions for QMNC improvement from women who gave birth during the two years of the COVID-19 pandemic.

**METHODS:**

Data were collected from an open-ended question included in a validated online questionnaire administered to mothers who gave birth in an Italian hospital between November 2020 to March 2022. The responses were analyzed using thematic analysis and mapped against the WHO Standards for improving QMNC and the WHO Framework of QMNC.

**RESULTS:**

The thematic analysis identified five main themes from the 2017 responses: 1) Support for mothers during the postnatal period; 2) Better use of resources; 3) Improvement of the maternity environment; 4) Reconsideration of organizational aspects; and 5) Guarantee of respectful practices. Women commented on all dimensions of the WHO framework except for two provision of care subdomains ‘actionable information and functional referral systems’.

**CONCLUSIONS:**

This is the first qualitative study in Italy focusing on women’s suggestions for improving QMNC during the COVID-19 pandemic. Its findings can be used to inform what aspects of QMNC need improvement in Italy. Collection of women’s views should be incorporated in routine monitoring of the QMNC, and data should be used for quality improvement purposes.

## INTRODUCTION

The WHO (2019) recognized quality of maternal and newborn care (QMNC) as a key determinant of maternal and newborn health outcomes, of health services expenditures, and a crucial aspect of application of human rights^1^. The service users’ perspective is pivotal for improving healthcare services as they can offer better insights on all the dimensions of QMNC provided including safety, efficiency, effectiveness, timeliness, equity, and people-centered care^2,3^. However, despite the need to include mothers and families’ perspectives within health services planning^4,5^, women’s voices and views on the QMNC received are not necessarily collected.

Several studies conducted in the pre-pandemic period^6,7^ offered insights on what women want in relation to QMNC across countries. In a recent study, Lazzerini et al.^8^ identified the following as key suggestions from Italian mothers for QMNC improvement: ensuring the presence of a companion of the woman’s choice during birth and throughout hospitalization, improving hospital spaces, increasing staffing, ensuring effective communication, professionalism, empathy, support, and enhancing newborn care information.

The COVID-19 pandemic significantly affected the QMNC, dictating reorganization of services, including introduction of restrictive infection-control measures and drastic changes in policies and protocols^9,10^. This unprecedented situation created serious challenges for both women, families and healthcare professionals (HCPs), and negatively impacted on maternal and newborn care globally at several levels, from health outcomes to service users’ satisfaction^10-12^.

An online cross-sectional survey conducted in Italy^12^, in line with international studies^13,14^, highlighted that women’s expectations and concerns about childbirth were significantly affected by the pandemic, with increased negative emotions, anxieties, fears, self-doubts and worries about health (especially of the baby and significant others) and concerns related to loneliness, compared to pre-pandemic experiences. However, there is still limited knowledge on women’s suggestions for improving QMNC at facility-level during the pandemic, especially in Italy.

The IMAgiNE EURO project used a validated online questionnaire, including 40 WHO Standard-based Quality Measures^15^, translated in several languages for assessing the QMNC at facility-level during and beyond COVID-19 pandemic. Mothers’ and healthcare professionals’ perspectives were sought for this purpose in more than 20 countries of the WHO European Region.

As a component of the IMAgiNE EURO Project, this study aimed at exploring women’s suggestions on how to improve QMNC in Italy, during the first two years of the pandemic. Though Italian data from the IMAgiNE EURO project have been previously analyzed^16,17^, this is the first study aimed at offering a thematic analysis of open-ended questions.

## METHODS

### Study design

A qualitative interpretive descriptive study^18^ was conducted using data collected via the Italian version of the IMAgiNE EURO survey. The study design and reporting adhered to the SRQR checklist (Supplementary file Table 1).

### Participants

Women aged ≥18 years, who gave birth in Italy from 1 March 2020 to 14 March 2022 were invited to participate in the IMAgiNE EURO online survey (Italian version). Women who gave birth in out-of-hospital settings were not eligible for participation. Recruitment was conducted through a setting-specific plan, utilizing social media, organizational websites, and local networks, including mothers’ groups and non-governmental organizations (NGOs). A total of 7928 women participated in the survey.

### Ethics

Ethical approval was obtained from the Institutional Review Board (IRB-BURLO 05/2020 15.07.2020) and conducted according to GDPR regulations. Prior to participation, women were informed of the study’s objectives and methods of the study, including their rights to decline participation; The privacy policy was made available for download prior to participation. Informed consent was obtained from each participant before they responded to the questionnaires. Anonymity was ensured by not collecting any personal identifiers. Data transmission and storage were secured through encryption to maintain confidentiality.

### Data collection

Data were collected from 18 November 2020 to 14 March 2022, using the IMAgiNE EURO validated online anonymous questionnaire and recorded using REDCap 8.5.21 (REDCAP, Vanderbilt University, USA; 2018) via a centralized platform. Participants received the questionnaire through the previously mentioned dissemination channels. For the purpose of this study, we analyzed specifically answers to the following open-ended question: ‘Do you have any suggestions to improve the quality of care provided at the facility where you gave birth?’. The answer to this open question was not mandatory and mothers could decide whether or not to offer their comments. Data were secured and accessible only to the research team.

### Data analysis

Descriptive statistics were performed to analyze the characteristics of the sample. A chi-squared test was used to compare the characteristics of the mothers who offered a comment on how to improve QMNC (our sample) to those who did not during the same study period.

An inductive thematic analysis based on the approach of Braun and Clarke^19^ was performed with the aid of NVivo12 software to all maternal responses. All texts included in the analysis were independently read, re-read and coded by two members of the research team (AN/SF), and preliminary units of meaning identified. A second coding iteration was undertaken by AN/SF/LI, with units of meaning clustered to form themes and subthemes; differences in interpretation were discussed to achieve agreement between researchers. The final coding was reviewed and agreed by all members who conducted the analysis.

Once identified, themes and subthemes were mapped against the WHO framework^2^ and the WHO Standards for improving the QMNC^20^. The WHO Standards for improving the QMNC is composed of three main domains: ‘experience of care’, ‘provision of care’, and ‘availability of physical and competent and motivated human resources’; comprising 8 Standards and 31 Quality Statements. Findings were also mapped in figures, to provide a visual representation of the contribution of maternal suggestions in relation to the areas of QMNC identified by the WHO Framework, in line with Lazzerini et al.^8^.

## RESULTS

### Women’ s characteristics

Out of the 7928 women participating in the IMAgiNE EURO survey in the Italian language, 2017 (25.4%) provided comments on how to improve the QMNC. Most respondents (1311/2017; 65%) gave birth in 2020, fewer in 2021 (632/2017; 31%) and a minority in early 2022 (65/2017; 3%). The sample characteristics are described in Table 1.

**Table 1:** Sample characteristics (N=2017)

*Characteristics*	*Categories*	*n (%)*
**Year of birth**	2020	1311 (65.0)
	2021	632 (31.3)
	2022	65 (3.2)
	Missing	9 (0.4)
**Maternal country of birth**	Italy	1922 (95.3)
	Other	95 (4.7)
**Maternal age** (years)	18–24	40 (2.0)
	25–30	409 (20.3)
	31–35	878 (43.5)
	36–39	499 (24.7)
	≥40	191 (9.5)
**Education level^a^**	None	0 (0.0)
	Elementary school	1 (0.0)
	Junior High school	70 (3.5)
	High School	743 (36.8)
	University degree	590 (29.3)
	Postgraduate degree/Master’s/Doctorate	611 (30.3)
	Missing	2 (0.1)
**Parity**	1	1467 (72.7)
	>1	550 (27.3)
**Type of hospital**	Public	1887 (93.6)
	Private	130 (6.4)
**Mode of birth**	Spontaneous vaginal	1250 (62.0)
	Instrumental vaginal	170 (8.4)
	Cesarean section	597 (29.6)
**Infant feeding**	Exclusive breastfeeding	1293 (64.1)
	Partial breastfeeding	616 (30.5)
	Formula	108 (5.4)
**Companion allowed to stay**	Always/nearly always	287 (14.2)
	Sometimes	451 (22.4)
	Never/almost never	1279 (63.4)
**Type of healthcare providers who directly assisted birth^b^**	Midwife	1740 (86.3)
	Nurse	508 (25.2)
	A student (e.g. before graduation)	104 (5.2)
	Obstetrics registrar/medical resident (under postgraduation training)	314 (15.6)
	Obstetrics and gynecology doctor	1129 (56.0)
	I don’t know (healthcare providers did not introduce themselves)	213 (10.6)
	Other	158 (7.8)
**Other conditions**	Newborn admission in ICU/SCBU	211 (10.5)
	Maternal admission in ICU	5 (0.2)
	Multiple birth	25 (1.2)

a Wording on education level agreed among partners during the Delphi. Questionnaire translated and back-translated according to ISPOR Task Force for Translation and Cultural Adaptation Principles of Good Practice.

b More than one possible answer. ICU: intensive care unit. SCBU: special care baby unit.

Supplementary file Table 1 compares characteristics of our sample with those of the mothers who did not answer the open-ended question. Nulliparity (p<0.001), public hospital as place of birth (p<0.001), instrumental vaginal birth (p=0.028), partial breastfeeding (p=0.013) and being assisted by practitioners who did not introduce themselves (p<0.001), were more frequent characteristics in the respondent group.

### Findings of the thematic analysis

Five main themes were identified: 1) Support for mothers during the postnatal period, 2) Better use of resources, 3) Improvement of the maternity environment, 4) Reconsideration of organizational aspects, and 5) Guarantee of respectful practices.

**Figure 1:**
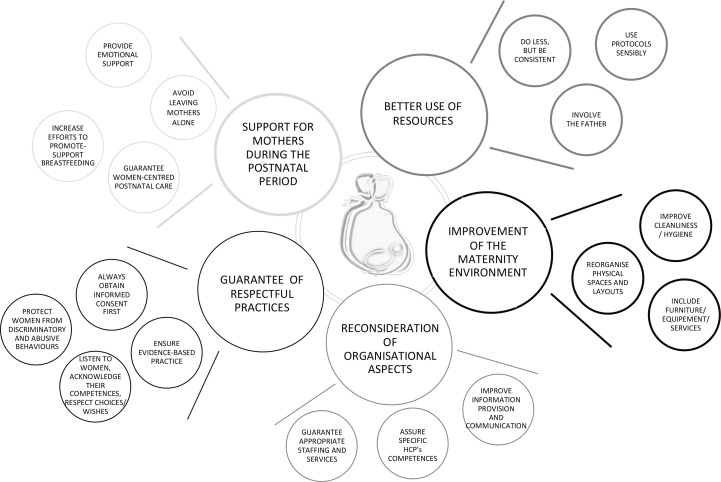
Themes and subthemes

Figure 1 illustrates the five themes with the related 18 subthemes, while examples of supporting quotes for each theme and subtheme are given in Table 2.

**Table 2:** Relevant supporting quotes for identified themes and subthemes and the corresponding WHO Standards and quality statements for improving the QMNC

*Themes*	*Subthemes*	*Supportive quotes*	*WHO Standards of care and quality statements*
**Support for mothers during the postnatal period**	**Guarantee women-centered postnatal care**	*‘Having the baby all the time with me in the room hasn’t allowed me to have even just an hour to rest, I had to beg the staff from the nursery that they could take him [baby] for 40 minutes so that I could have lunch and a showe*r.’ (W4563) *‘It is inconceivable that, after a cesarean section, a woman is [considered to be] able to get up to take her baby, breastfeeding him, changing his nappies, take him to the nursery for the [medical] visits, as well as washing herself and eat all by herself and by the time the staff comes to take away the tray as they are ending their shift.’* (W11328) *‘To avoid discharging 48 hours after a cesarean section, to discharge when milk arrives to avoid returning 36 after the discharge to check the baby.’* (W9659) *‘Guaranteeing early discharge after birth and midwifery home visiting after 24 hours.’* (W55) *‘The staff should have tried, at least, to make up for the lack of a family [supportive] network.’* (W8794)	**Standard 1** Quality statement 1.1c: Mothers and newborns receive routine postnatal care.
	**Avoid leaving mothers alone**	*‘Dad’s presence for all the time the mum would need, including the night. Don’t leave mums alone!’* (W9901) *‘Ιt would have been important to me that my partner could have accessed the hospital, as well as my older kids.’* (W7777) *‘Longer visiting time and access for more than one person. Maybe one person at the time, but parturients need help after birth and they need their family.’* (W3350) *‘A new mum needs someone next to her that helps her or that simply listens to her, the workers were presents and understanding, but they are not the family.’* (W6877) *‘It would be sufficient to do a swab test [ for COVID-19] to enable to prolong visits.’* (W443) *‘ …we were available to get the swab tests even privately.’* (W892) *‘During my staying there were no visits due to COVID-19 measures (except for the husband’s visit 2 hours a day). It has been really positive to recover and to bond with the newborn.’* (W7730) *‘The lack of visits during the staying was very positive to me, conversely I missed a lot the dad’s presence [partner]*.’ (W502)	**Standard 1** Quality statement 1.1a: Women are assessed routinely on admission and during labor and childbirth and are given timely, appropriate care. **Standard 6** Quality statement 6.1 Every woman is offered the option to experience labor and childbirth with the companion of her choice. Quality statement 6.2: Every woman receives support to strengthens her capability during childbirth.
	**Increase efforts to promote and support breastfeeding**	*‘Better work is necessary as regards breastfeeding care. It [breastfeeding] is too precious and important.’* (W10580) *‘Greater attention to breastfeeding, with adequate support.’* (W124) *‘A dedicated figure for breastfeeding supports should be identified.’* (W5148) *‘A breastfeeding counsellor should never lack in a hospital organization and should help the new mum since the very first instants after birth.’* (W7409) *‘To have more facts and less words on breastfeeding.’* (W5252) *‘When a midwife is called to help a mum to attach the baby, she should teach he mum how to do it autonomously and not fitting herself the nipple in the baby’s mouth, this doesn’t make mum autonomous nor competent.’* (W7642) *‘Many mums haven’t continued with breastfeeding as poorly supported during hospitalization.’* (W230) *‘I deemed them [HCPs] to be partly responsible for my breastfeeding failure and the situation has certainly influenced a lot on my subsequent postnatal depression.’* (W6857) *‘Or, if rooming-in is in place, make sure that the woman has all the help and support to look after the neonate.’* (W10987) *‘Be more flexible with rooming-in!’ (*W2439)	**Standard 1** Quality statement 1.1c: Mothers and newborns receive routine postnatal care.
	**Provide emotional support**	*‘Given the moment of solitude I would have appreciated the presence of a psychologist to know how I felt in the postpartum. The solitude experienced by parturient during the pandemic hasn’t been much considered. Fortunately, I hadn’t particular difficulties but I could have had.’* (W11844) *‘Emotional support is necessary. Given the staff overload, I reckon that it should be employed perinatal psychologists for this objective, beyond the pandemic.’* (W4097) *‘Greater psychological support and attention to mothers who gave birth to be with her baby, due to admission to NICU.’* (W11399)	**Standard 1** Quality statement 1.1c: Mothers and newborns receive routine postnatal care.
**Better use of resources**	**Do less, but be consistent**	*‘Ensuring one to one care.’* (W9955) *‘Concerning privacy, at birth I had a big audience with the midwife, the doctor and registrars … privacy zero.’ (*W3638) *‘During my staying there was a too high turnover and each time I had to start again to recap on my baby’s needs.’* (W3483) *‘The healthcare workers didn’t wear a badge with their name and role, I had no idea whom I was talking too.’* (W4593)	**Standard 1** Quality statement 1.1a: Women are assessed routinely on admission and during labor and childbirth and are given timely, appropriate care.
	**Involve the father**	*‘Fathers do exist. COVID has erased them!’* (W8565) *‘Dads should have more rights. My husband couldn’t even hold her baby! It’s a shame!’* (W2744) *‘Having your partner with you is necessary to give birth.’* (W6928) *‘I will never give birth alone again! If they won’t allow fathers to be with us from the beginning of labor, the next child’s birth will occur at home or in a maternity home* [birth center].*’* (W950)	**Standard 6** Quality statement 6.1: Every woman is offered the option to experience labor and childbirth with the companion of her choice.
	**Use protocols sensibly**	*‘Hospital protocols have been applied in a totally illogical manner, causing further stress and unease.’* (W931) ‘It’s a no-sense to accept parturients [to be in hospital] and not the partners they live, sleep with etc.*’* (W325) *‘A 4-hour waiting for a swab results it too much … they should improve those times as for that reason my husband could not be at birth as it took me less than 4 hours to give birth.’* (W5354)	**Standard 1** Quality statement 1.9: No woman or newborn is subjected to unnecessary or harmful practices during labor, childbirth and the early postnatal period.
**Improvement of the maternity environment**	**Reorganize physical spaces and layout**	*‘The situation in the ward is like in the Third World: there are not enough toilets; they are not frequently cleaned; the building is dilapidated; there is no privacy.’* (W7686) *‘My room was really far from the nursery and, having undergone a cesarean, to make all that route to have some advice was uncomfortable. I would move the rooms of those who had a cesarean close to the nursery.’* (W10893) *‘To provide and equip a space where also the other kids can come and visit you.’* (W12029) *‘It’d be appropriate to direct COVID-patients in dedicated structures so to avoid creating further confusions and tensions for all in such a delicate moment.’* (W6851) *‘To me sharing the same room with another woman after birth is inconceivable, COVID or not-COVID there should be smaller but single rooms.’* (W8646) *‘Soundproofing all labor wards so that you can’t hear the other laborers.’* (W545) *‘Inadequate ventilation in the rooms!* … *Constant cold air, day and night* … *and you couldn’t control it, even if the thermostat was there it wasn’t functioning.’* (W4520) *‘When I managed to have a bed, it was not in the maternity area but in the gynecology ward. 8 mums had to be in gyne with me, at the opposite side of the nursery because of bed shortage. We had to exit the ward, cross all the outpatients and common areas where there were hundreds of non-admitted people, exposing ourselves and our babies to high risks of contagion. In the gyne ward there is only one bathroom to share.’* (W8747)	**Standard 8** Quality statement 8.2: Areas for labor, childbirth and postnatal care are designed, organized and maintained so that every woman and newborn can be cared for according to their needs in private, to facilitate the continuity of care.
	**Improve cleanliness and hygiene**	*‘To improve hygiene in hospitals.’* (W1331) *‘To improve cleanliness of rooms and bathrooms.’* (W9377) *‘To guarantee hygiene and cleaning practices in rooms and bathrooms, no more [performed] in a superficial manner but in depth with disinfection of all areas and parts, as it’s inconceivable that a public hospital building is the source of germs and bacteria.’* (W7500)	**Standard 8** Quality statement 8.1: Water, energy, sanitation, hand hygiene and waste disposal facilities are functional, reliable, safe and sufficient to meet the needs of staff, women and their families.
	**Include furniture or equipment and services**	*‘Providing rooms with changing tables.’* (W6505) *‘ … and wardrobes to put all that the parturient needs, the luggage, the bags.’* (W6239) *‘Profile beds with banks are fundamental in my opinion.’* (W7127) *‘To improve the hospital staying with an internet connection and/or simply by putting a television.’* (W6960) *‘There was no television in the room. I know is not fundamental, however during the hospitalization it hasn’t been possible to be visited even by my husband and days looked infinite. Perhaps a television could have helped.’* (W1818) *‘Devices for wireless monitoring [CTG] that permit [women’s] movement.’* (W9034) *‘Due to the pandemic, the labor room with the birthing pool was converted to an assessment room, so waterbirth wasn’t possible. As the pandemic continues it would be nice to restore the pool.’* (W7190)	**Standard 8** Quality statement 8.3: An adequate stock of medicines, supplies and equipment is available for routine care and management of complications.
**Reconsideration of organizational aspects**	**Guarantee appropriate staffing and services**	*‘To improve the care in the hospital I gave birth in, additional staff should be employed so to guarantee an adequate workload [to professionals] for the safety of patients, newborns and of the personnel themselves.’* (W10928) *‘The number [ of staff] is not sufficient to meet the inpatients’ needs and necessities.’* (W255) *‘The staff is much under stress … the stress transpires more than the willingness to help women … this is an indicator of the necessity to increase the staffing to alleviate the situation.’* (W2995) *‘More staff is needed as it’s evident that the existing one is too scarce and reduced to make exhausting shifts that worsen the quality of the work.’* (W11466) *‘I believe that, in general, there should be more funding for public healthcare.’* (W10542) *‘More investments in the healthcare system is needed, the staff was in grand part extremely valid yet extremely undersized.’* (W11274)	**Standard 7** Quality statement 7.1: Every woman and child has access at all times to at least one skilled birth attendant and support staff for routine care and management of complications.
	**Assure specific HCPs’ competences**	*‘Too young and inexperienced [healthcare] workers don’t help the quality of the service … too young and unprepared midwives, at times even arrogant.’* (W6984) *‘Too many students left without a true guide. To learn in a university hospital is right but not to the detriment of patients.’* (W8676) *‘Less competition amongst doctors!’* (W10154) *‘Clinical competence is not enough.’* (W11936) *‘More competence and compassion/cordiality.’* (W6233) *‘More human touch for certain healthcare workers, we are not numbers!’* (W10218) *‘Who [amongst HCPs*] *is afraid of COVID should stay at home, and who hasn’t empathy for those they should care of, change job.’* (W4209) *‘There was such a disproportionated discrepancy between the kindness of some midwives and obstetricians and the rudeness and unkindness of others.’* (W7036) *‘Improving staff training on breastfeeding and support to new-mothers is definitely needed.’* (W230) *‘Training the personnel on empathic and respectful communication.’* (W188) *‘Teaching to the nursery staff a little bit of empathy and how to use language with a new-mother.’* (W8410) *‘The only suggestion I have is that they should teach empathy to the hospital staff!’* (W7802) *‘To select the healthcare personnel also based on human and psychological criteria.’* (W10569) *‘Because it’s better that a doctor without heart and gentleness would devote themselves to do something else in life.’* (W2722) *‘Avoiding letting professionals without hearts but ice in their place to enter these sites [hospital], they are not doing any good to those who give birth.’* (W9913)	**Standard 7** Quality statement 7.2: The skilled birth attendants and support staff have appropriate competence and skills mix to meet the requirements of labor, childbirth and the early postnatal period.
	**Improve information provision and communication**	*‘Provide more information.’* (W11216) *‘Be clearer on what is going on. Additionally, my baby’s health situation wasn’t explained properly.’* (W10951) *‘Improving the information provision to family and husband … keeping them informed.’* (W7188) *‘Tell the truth on what is happening and explain what they are doing!’* (W8703) *‘When performing a scan, I would plea the personnel to communicate with us instead of staying silence for the duration of it, with no comments, no showing the screen.’* (W10827) *‘When I arrived [to the ward] after isolation, I have been left alone without receiving any information on how the ward works (for example what where my spaces and where to find everything to change my baby’s nappies).’* (W7632) *‘Little information and confusing and conflicting at times, depending on the worker you were speaking with.’* (W7373) *‘Midwives and pediatricians should give the same, and not contrasting between each other, information.’* (W8664) *‘To improve information provision and communication during handovers and possibly in the patient’s presence.’* (W344)	**Standard 4** Quality statement 4.1: All women and their families receive information about the care and have effective interactions with staff. Quality statement 4.2: All women and their families experience coordinated care, with clear, accurate information exchange between relevant health and social care professionals.
**Guarantee of respectful practices**	**Ensure evidence-based practice**	*‘They [HCPs] should keep in mind that there are guidelines to abide by.’* (W5203) *‘To abolish the maneuver of [fundal] pressure on the belly.’* (W4362) *‘Avoiding Kristeller and being in hurry [waiting not] during birth!’* (W10157) ‘R*educing the number of inductions without a true reason of danger for the baby.’* (W230) *‘They should not perform a cesarean unnecessarily!*’ (W11359) *‘All settings should permit free movement and positions in the expulsive phase of birth instead of resorting to pharmacological stimulation [augmentation] and episiotomy*.*’* (W5547) *‘I would suggest to delay cord clamping of a few minutes.’* (W10859) *‘All midwives should guarantee skin-to-skin moments in the first 2 hours after birth.’* (W11402) *‘I suggest urging [maternity] services to promote more breastfeeding but most of all the gentle cesarean practice (where possible) where mum and baby’s right to have skin-to-skin is protected so as the husband’s presence in operating theatre.’* (W4297) *‘When my babies was born she was full of amniotic fluids in the lungs and because she was breathing normally they did not perform suctions, but please do suctions always even if it is invasive as when a mother sees her just-born daughter becoming purple 3 times and stopping breathing and ‘vomiting’ water… it’s scaring and I was lonely and terrified, but the personnel has been wonderful with me thanks.’* (W800) *‘Once, they performed suction at birth, but it seems invasive nowadays. To me, seeing your baby with eyes like ping-pong balls and all purple and not breathing is worse … so I only suggest that, to restore it [suctioning], as for everything else you are fantastic.’* (W8424)	**Standard 1** Quality statement 1.9: No woman or newborn is subjected to unnecessary or harmful practices during labor, childbirth and the early postnatal period.
	**Always obtain informed consent first**	*‘The day before my birth I was assessed by a female obstetrician (who saw me for the first time) who decided to perform membrane sweeping (in addition painful) as final attempt to make labor starting spontaneously without telling me or asking me permission … and all this to avoid the cesarean scheduled for the day after.’* (W2217) *‘They administered formula milk top-ups to my baby without my consent.’* (W5317) *‘It should be mandatory to explicitly ask for authorization before performing interventions such as breaking waters or episiotomy and the motives for doing it should be clarified first.’* (W9564) *‘Consent to be asked before using synthetic oxytocin on parturients.’* (W1382)	**Standard 5** Quality statement 5.3: All women have informed choices in the services they receive, and the reasons for interventions or outcomes are clearly explained.
	**Listen to women, acknowledge their competence and respect choices and wishes**	*‘Trying to listen more to women’s wishes and to baby’s needs.’* (W6809) *‘Respect for woman’s self-determination and choice of the birth she wants.’* (W11217) *‘Remind them [HCPs]* t*hat they are dealing with sentient human beings [women] that must be able to choose what is best for themselves!’* (W2594) *‘I think that our instinct as mothers and of the baby we carry should be respected more.’* (W10152) *‘In antenatal ward they should listen more to women and not undervalue mums’ symptoms and sensations*.*’* (W3240) *‘Let the woman listen to herself, her body and behave accordingly, and intervene as little as possible.’* (W9797)	**Standard 6** Quality statement 6.2: Every woman receives support to strengthens her capability during childbirth.
	**Protect women from discriminatory and abusive behaviors**	*‘That decision [of not breastfeeding] should be respected and those women treated and supported like the others.’* (W9401) *‘Because I had [private] insurance, I was cared by my midwife and obstetrician throughout the entire continuum, receiving a complete care, including having epidural guaranteed, the possibility of my husband to be with me in labor ward and postnatal ward with a single room (and an additional bed for him) all the time. All women should have the same possibility of living birth in this way and not only those who can afford it.’* (W7379) *‘In my case, differences [in behavior and practices] were made because my partner and I were not vaccinated for SARS- CoV-2.’* (W9664) *‘I discovered to be positive for COVID after birth during hospitalization, and the level of care after ascertain my positivity has shockingly diminished. I have been treated as a plague-ridden.’* (W1944) *‘Because of being COVID-positive we have been forced to give birth in a different city from home, with no one that could stay with you.’* (W8874) *‘To avoid discriminatory phrases towards those who have some mental health issues.’* (W2412) *‘Do not make differences between foreigners and Italians, we are all the same.’* (W10109) *‘And most of all there should not be prejudices towards those who don’t have the right height and weight measures.’* (W10305) *‘The habit of calling you “mum” [not with your name] is highly depersonalizing. Many of my acquaintances felt the same.’* (W7456) *‘Aloud offenses, pressures and else to whom doesn’t choose the breast [to breastfeed].’* (W4560) *‘I was terrified and nurses were making fun of me because I was crying.’* (W1292) *‘A nurse intimidated me to sush and do not disturb.’* (W4408) *‘They leave you on the bed with your legs open without being covered, violating your intimacy and dignity as whoever enters the rooms can see everything.’* (W680) *‘I felt as meat for slaughter.’* (W2046) *‘Avoiding threatening people (the midwife threatened of leaving me during labor if I wouldn’t have breathed in the ‘right’ way.’ (W1598)* *‘Do not practice obstetric violence!’* (W4151) *‘Obstetric violence does exist and I was a victim as many other women. It marks you in an indelible way and will accompany always the memory of my daughter’s birth.’* (W8493) *‘There should be a law against obstetric violence.’* (W9993) *‘Heavy sanctions, with termination of contract, in case of mistreatment of patients.’* (W8891)	**Standard 5** Quality statement 5.1: All women and newborns have privacy around the time of labor and childbirth, and their confidentiality is respected. Quality statement 5.2: No woman or newborn is subjected to mistreatment, such as physical, sexual or verbal abuse, discrimination, neglect, detainment, extortion or denial of services.

**Figure 2:**
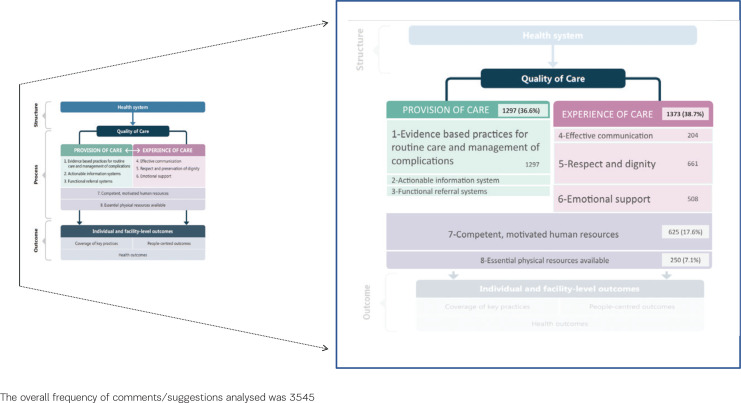
Mapping of findings against the WHO framework for QMNC: contribution of participants' suggestions to WHO domains and standards

Figure 2 shows the result of the mapping of findings against the WHO framework^2^ and the WHO Standards for improving the QMNC^20^, and the different contribution of participants’ suggestions to its domains. The highest number of comments related to the domain of ‘experience of care’ (n=1373; 38.7%) followed by the domain of ‘provision of care’ (n=1297; 36.6%) – and fully regarding the Standards of ‘evidence-based practice’ – then by the domain of ‘competent, motivated human resources’ (n=625;18.1%) and ‘essential physical resources available (n=250;7.3%). The only aspects of the WHO framework that appeared not represented in women’s comments were two subdomains from ‘provision of care’: ‘actionable information system’ and the ‘functional referral system’.

### Support for mothers during the postnatal period

*Guarantee women-centered postnatal care*

Women highlighted a disparity between antenatal/intrapartum care and care received after birth denouncing a significant reduction of the QMNC in the postpartum period. According to participants, postnatal care was mainly focused on the newborn, leaving mothers with a sense of abandonment. Women experienced a lack of support even addressing basic physiological needs such as the possibility of using the toilet, sleeping or eating.

Women’s experiences led them to suggest the need for guaranteeing true women-centered postnatal care at all levels and processes, including hospital discharge, and greater attention to women’s individual needs, especially for mothers with specific conditions [e.g. after cesarean section (CS) with the baby being admitted to the neonatal intensive care unit (NICU)].

The respondents stated that support from healthcare professionals (HCPs) was even more crucial during the pandemic, as women could not receive help from their significant others during hospitalization due to the restrictive measures adopted during the provision of maternity services.

*Avoid leaving mothers alone*

Women advocated for the unrestricted presence of their companion of choice during hospitalization. The participants indicated a need to adopt more flexible policies for visits, with suggestions ranging from just extending visiting time to guaranteeing that companions may stay for as long as the women wished. A number of women requested that this flexibility be applied to any family member, stressing the unique role of family support in a crucial moment of life such as birth.

However, some women offered counterviews and suggested restricting visits during hospitalization, provided that fathers could have free access. Limiting visitors is associated with greater opportunity to rest, have a quiet time and bond with the baby.

*Increase efforts to promote and support breastfeeding*

Numerous comments focused on the need for better breastfeeding support, especially in the immediate postpartum period. These suggestions highlighted women’s awareness of the benefits of breastfeeding for both mothers and babies and the crucial role of HCPs in promoting it. Women generally acknowledged the benefit of rooming-in but requested a more women-friendly and flexible translation of the model into practice.

Some participants advocated for specialist figures, e.g. International Board of Certified Lactation Consultants (IBCLCs), to be routinely included in the maternity staff to foster positive experiences of breastfeeding.

The respondents indicated the need for HCPs to avoid hands-on practices while supporting women and to provide timely and practical information and advice. Women wanted a kind of support that could increase their self-efficacy and competence and enable them to feel more confident in self-assessing whether the baby was breastfeeding well and safely.

The most requested technical information was how to breastfeed in different positions; according to the participants, information should be provided through practical courses delivered by experienced staff members and not just through brochures or videos.

*Provide emotional support*

Suggestions concerning emotional wellbeing were also received. The loneliness experienced by many participants during the pandemic seems to underpin many of the comments analyzed. Moreover, women advised that there was a clear need to involve a specialized professional (e.g. a psychologist or a counsellor) who can help them with their emotional and psychological needs immediately after birth and guarantee their presence to alleviate the loneliness women experience, which was more prominent during the pandemic. Emotional support is particularly recommended for women who undergo CS or are separated from their newborn that needs admission to the NICU.

### Better use of resources

*Do less, but be consistent*

Women felt stressed by the lack of consistent care by the same midwife due to excessive professional turnover during shifts. They recommended the provision of one-to-one support and individualized midwifery care to all women. Continuity of care was indicated as a key element in improving women’s birthing experiences. There were also specific suggestions on limiting the number of HCPs present at birth to ensure privacy.

Women asked for all HCPs encountered to introduce themselves and specify their professional background and role to meet the needs of the mother and the newborn.

*Involve the father*

Women defined the impact of the restriction of fathers from being present at birth, due to the COVID-19-induced restrictive measures in hospitals, as not only a ‘denied experience’ but also a ‘denied right’ for their partners. They wanted their partners to be directly involved in childbirth as a protagonist, who would actively participate in the ‘unique’, ‘exciting’ experience of seeing their baby come into the world. Women reported that frequently the father’s first encounter with their baby was delayed, and the transition to parenthood was ‘deferred’ and postponed to the moment of family reunification at home. It resulted in fathers experiencing a sense of ‘missing out’. For this reason, women recommended the father’s presence from early labor and for as long as the family wanted. Participants indicated how vital the supportive presence of their partner was in meeting their physical and emotional needs. In some cases, the absence of partner involvement led to women experiencing negative feelings that had long-lasting negative emotional consequences.

*Use protocol sensibly*

Women highlighted how the restrictive measures adopted in hospitals in response to the pandemic negatively impacted their birth-giving experience. They recognized the need for interventions and reorganization of services and policies to reduce the risk of COVID-19 infection while wishing for greater flexibility in protocols and a more compassionate, reasonable, women-centered approach. Examples included the long time needed to process the partner’s nasal/ oropharyngeal swab test results, which limited their presence and support. The participants suggested that offering COVID-19 rapid antigen tests to partners would have improved the overall parental experience and the quality of bonding with the newborn.

### Improvement of the maternity environment

*Reorganize physical spaces and layouts*

The participants also commented on the physical environment. Some women reported giving birth in a ‘crumbling’ and ‘outdated’ environment and suggested there was a need to renovate maternity care facilities to enhance safety and to better fulfil women’s needs. Participant comments frequently featured requests for an environment conducive to privacy, comfort, dignity, and respectful and compassionate care. According to the participants, maternity spaces must be organized around specific and different maternal clinical conditions and health needs. Examples included dedicated rooms for mothers whose babies were in the NICU, separate wards near the nursery for women who had undergone CS, and social areas where mothers and relatives/visitors could spend quality time together. Women also requested dedicated paths, or even full areas or hospitals, for COVID-19-positive women. With regard to the specific features of rooms, suggestions ranged from ensuring the availability of single rooms – preferably with an ensuite bathroom – to sound-proofed walls, soft lighting, and adequate temperature control.

*Improve cleanliness and hygiene*

Some comments reported a low standard of cleanliness and hygiene inside the maternity unit, leading to suggestions of adequate sanitation, hygiene and infection prevention as integral parts of QMNC. The cleanliness of bathrooms and maternity rooms appeared crucial to ensure a safe environment for the newborn and the mother.

*Include furniture or equipment and services*

The participants described the need for functional furniture, e.g. personal wardrobes, profile beds with appropriate side tables, comfortable chairs for breastfeeding and mosquito nets, to be provided in all ward rooms. The women also suggested improving the quality of soaps, toilet paper, and hand towels. Wireless cardiotocographs and electric breast pumps were mentioned among the technological equipment that can be used to ensure more women-centered care.

Some comments highlighted how the COVID-19 pandemic also impacted access to previously available furniture or equipment, such as birthing pools, which the women wanted to be accessible on request at all times. New resource needs were associated with the pandemic, such as the provision of appropriate types and quantity of face masks. Moreover, the availability of a Wi-Fi connection and a television was deemed necessary to reduce the perceived loneliness and isolation due to the restrictions.

### Reconsideration of organizational aspects

*Guarantee appropriate staffing and services*

The women deemed the current number of staff members to be absolutely inadequate, especially during night shifts, weekends and holidays, to the detriment of QMNC. The participants strongly advocated for greater investments in human resources and asked for governors’ responsibility and commitment in securing public funds to address staffing issues. The comments acknowledged that excessive workload and burnout were the main elements affecting HCPs.

With regard to services, the women recommended improvements to catering services in order to properly address mothers’ nutritional needs by enhancing the quality of meals and ensuring a sufficient amount of water supply to support hydration during hospitalization.

*Assure specific HCPs’ competences*

The participants stated that the availability of trained, competent, humble and emphatic HCPs to care for them and their families was crucial. Some women reported a high prevalence of younger professionals, possibly with limited experience. In particular, they expressed concerns regarding professionals under training (i.e. registrars). Interprofessional competitiveness and the fear of working during the pandemic were clearly perceived by the women. The lack of compassionate, women-centered, humanized care emerged in several comments and led women to suggest that qualities such as kindness, empathy and active listening be included amongst the criteria for recruiting or selecting personnel.

The participants clearly identified the following areas as priorities during staff training: evidence-based practice to promote and support breastfeeding, and respectful and empathic communication, including active listening.

*Improve information provision and communication*

Suggestions also covered the area of communication and information provision. Women advocated for greater attention to communication about mothers’ and babies’ health with clear, understandable and truthful information.

The participants expressed the need for more information regarding clinical procedures and the organization of services, and recommended the integration of verbal communication with other informative resources (e.g. videos and brochures).

Comments also highlighted issues around conflicting messages from different professionals and among various maternity department protocols, indicating the need for improving the communication among departments, HCPs and women.

### Guarantee of respectful practices

*Ensure evidence-based practice*

Women expressed a strong desire to the cessation of any non-evidence-based practices for both mother and baby, such as membrane sweeping, Kristeller manoeuvre or the induction of labor, and episiotomy when performed without clear indications.

Suggested practices to perform included an active offer of vaginal birth after cesarean (VBAC), epidural, mother–baby interaction and skin-to-skin contact in the operative theatre during CS, freedom of movement in labor, choice of birth position, delayed cord-clamping, and others practices that promote newborns’ adaptation to extrauterine life (e.g. skin-to-skin contact).

Some women’s suggestions included practices that they believed to be evidence-based, although this was not necessarily the case (e.g. the suctioning of the baby’s airways at birth).

*Always obtain informed consent first*

Obtaining women’s full informed consent before the commencement of any procedure emerged as a must-to-implement change. Several comments reported negative experiences related to clinical interventions performed without appropriate/full consent.

Several participants stressed how informed consent should be obtained for all procedures, especially during labor and birth. The women emphasized the importance of thorough, evidence-based information, including indications, benefits and risks of a proposed intervention, to enable true informed consent. Some participants flagged specific areas of improvement, such as infant-feeding care, where parental authorization should be obtained before any formula feeding or nutritional supplement administration to the infant.

*Listen to women, acknowledge their competence and respect choices and wishes*

A lack of active listening by HCPs seemed to underpin women’s recommendations related to respecting their choices and wishes, avoiding judgments and acknowledging their competence in care. Areas where they perceived their competences to be particularly overlooked or unsupported and their voice and wishes to be unheard included pain management, mode of birth and breastfeeding. The women wanted HCPs to recognize and promote maternal competence, suggesting the elimination of unnecessary and disempowering interferences and greater trust in women’s ability.

*Protect women from discriminatory and abusive behaviors*

In addition to the need for HCPs to value women’s competences, the participants commented on the necessity to change practices that actively result in women’s sense of inadequacy or, even worse, appear as mockery, judgments or insults related to their choices or personal characteristics. A plea to protect women from any disrespect and abuse in maternity care emerged from the accounts of verbal abuse, including offences, threats and invitations to ‘shush’, as well as physical violence such as being kept in a position for birth different from the woman’s choice or application of the Kristeller’s manoeuvre.

The elimination of all possible forms of discrimination was also strongly requested by women. The participants emphasized the need to always be treated with equity and kindness during care. In this respect, the women highlighted the disparities between public and private services, indicating discrimination based on personal financial status. Some participants acknowledged themselves as ‘privileged’, as they could afford private care that provided better quality treatment and positive experiences. Others reported being influenced by their choices in relation to vaccination for COVID-19 or their status or physical characteristics, such as being a foreigner or obese.

The participants also commented on the significant long-term impacts of experiencing disrespect and/or abuse on their wellbeing. They denounced being hardly recognized, at times, as victims of disrespectful practices, as the latter seemed to be embedded as normal practices in the health system culture. The women called for this issue to be properly addressed by means of legislation and asked for fines and disciplinary actions to be considered in cases of any reported abusive behavior from HCPs during hospitalization.

## DISCUSSION

This is the first qualitative study exploring women’s suggestions for implementing QMNC during the COVID-19 pandemic, in the Italian language. The study involved a large sample of responders, providing many inputs for improving the QMNC, suggesting that mothers are ready to provide their views, and that their ideas are valuable.

Women’s strong request for implementing support and care during the postnatal period reiterate needs that emerged since the pre-pandemic time. These include the desire to feel adequately cared for, to be seen as individuals, with physiological and emotional needs^21,22^. Moreover, our findings confirm disparities in the QMNC perceived postnatally compared to the provision of antenatal and intrapartum care, aggravated by the pandemic for the presence of restrictive measures and reorganization of services^22,23^. Most of the postnatal experiences shared by women support their suggestions, reflect what emerged from other qualitative studies conducted during the pandemic, and reveal transversal issues concerning QMNC across countries. For instance, women’s exacerbated loneliness due to diminished (or even removed) access to support networks, sense of isolation, feeling of abandonment and disempowerment without the partner, appeared as some of the key challenges that participants in our study shared with international peers^24,25^.

The need for implementing breastfeeding support, based on women’s negative experiences of its care, also appears in line with previous research conducted in European countries during the COVID-19 outbreak^16,24^. The worrying levels of stress and mental health issues related to breastfeeding during the pandemic are known^26^. The requests for a dedicated professional, e.g. psychologist in the team, show how critical this aspect of postnatal care was perceived by women. Furthermore, the calls for guaranteeing other specialist figures such as IBCLC, stress once again that needs voiced before the pandemic, including the request for greater practical support for breastfeeding^27^, were not necessarily heard. Women’s comments on the co-existence of nurseries and rooming-in also show the persistence of known barriers to this evidence-based practice in the Italian context^27^.

In our study, women reported a lack of support and a poor translation of the rooming-in model into practices. This led to a desire of returning to nursery systems, understanding the benefits. Interestingly, a similar finding was reported during pre-pandemic time, and in line with other studies^28^ from WHO European Region countries. Moreover, such a result challenges existing claims of a strengthening of breastfeeding and family-friendly policies and support – including rooming-in – during COVID-19 in Italian hospitals. Nevertheless, in other studies, negative experiences were related to opposite situations, i.e. mother’s separation from the baby during the pandemic, though the common ground could be represented by organizational issues^29^.

The participants’ request for greater consideration and involvement of fathers/partners in the care pathway amplify similar pleas emerging both before and during the SARS-CoV-2 virus spread^23^. Women’s comments gave louder voice to denied experiences, sense of ‘missing out’ and being forgotten and negative consequences that fathers/partners underwent during the pandemic^23,30^. This emotional/experiential loss does not appear to be addressed by current health services so far^23^ and certainly requires to be given attention as a public health issue.

It is worth noting that from women’s perspective, even the best HCPs’ care could not make up the missed presence and support from their companions of choice and/or family and friends during the childbirth journey. These being two distinct types of support, addressing different needs and human rights to guarantee^23,24^, hence not replaceable or interchangeable, regardless of the circumstances.

The inconsistencies in companionship and visiting policies in maternity services and recommendations to adopt a more flexible approach reflect previous discussions. Some unexpected views on benefits of visiting restrictions in our study also resonate with previous research^24,31^.

The urgency to act on poor staffing levels seemed further exacerbated by the strain that the pandemic placed on maternity systems^22,32^. Notably, the crisis created by the pandemic did not make poorer QMNC related to staffing level more understandable, nor acceptable to women. This cannot be ascribed to women’s lack of recognition of the extra pressures and challenges faced by HCPs as this was evident globally^23^, but rather to an awareness of their needs and rights. Our findings contribute in clarifying that women, though considering staff numbers insufficient, are looking at the issue beyond a numeric perspective and advocate for staffing professionalism that can guarantee appropriateness of care. In this sense, appropriateness was also related to securing continuity of care and core HCPs’ competencies, qualities, including communication skills and compassionate approach, which significantly affected the QMNC during and beyond the pandemic^16^. Many HCPs recognized the negative impact of the pandemic on the care provided and their skills mirroring mostly women’s views^33^. However, counter-experiences where Italian HCPs felt some of their skills improving during COVID-19, and women reporting provision of kind and competent care, are also reported in the literature^34^.

Furthermore, suggestions about the abandonment of disrespectful practices reflect the violation of women’s rights, including lack of informed consent prior to procedures, abusive behaviors, and conscious and unconscious biases prior and during the pandemic^29,35^ were not adequately dealt with. The national and international debates, recommendations, framework and calls for enhancing respectful maternity care and eliminating disrespectful and abusive practices of the last decade^2,6,36,37^, appear insufficient to remove structural dimensions^38^ that maintain those practices, warranting further actions and innovative approaches to counteract them.

Greater consideration of the maternity environment was also indicated as an intervention to prioritize for implementing the QMNC in hospitals. Our findings confirm the importance of well-known elements such as cleanliness of maternity settings, adequate and woman-centered equipment that foster women’s choices, a spatial layout that promotes privacy and support biopsychosocial birth processes^20,39^.

Our study highlights that a large proportion of the respondents were nulliparous (72.7%), indicating that this was their first childbirth experience. This finding is significant because the experiences of these first-time parents during the COVID-19 pandemic could have important implications for the future. The isolation and high stress levels associated with giving birth during this period may influence their future decisions regarding childbirth. As healthcare professionals, it is crucial to recognize and address these traumas, to provide adequate support in subsequent pregnancies.

Finally, unlike the findings from previous research by Lazzerini et al.^8^, adopting the WHO framework and standards to evaluate women’s views on how to implement QMNC, our participants’ comments pertained to the WHO ‘experience of care’ domain as much as the ‘provision of care’. Women’s awareness of key components of QMNC may have increased during the pandemic time due to a number of needs being further unmet during this time. Women did not directly comment or suggest actions on the ‘referral and actionable information systems’ component of the WHO framework, representing the only uncovered area. This may be explained by the nature of this domain’s indicators/measure. Other stakeholders, e.g. HCPs, may represent a more appropriate data source for investigating this type of information. The ongoing data analyses on the surveys involving HCPs within the IMAgiNE EURO project, may thus enable greater insights in the future.

### Strengths and limitations

The main strength of this study is that it offers a better understanding of women’s experiences during the pandemic and provides clear direction on priority areas for better QMNC. Although a large sample size is not per se an element of better quality in qualitative research, as argued by Eri et al.^24^, analyzing such a large number of answers to open-ended questions provided extensive insights into women’s experiences, views and suggestions. In this sense, we feel that the richness of the insights offered by our participants was not necessarily proportionate to the wordiness of their comments and some concise answers were as effective as more detailed ones in indicating aspects of care that worked or to implement. Future research could contribute to exploring the experiences in different birth settings or specific population sub-groups experiences (e.g. vulnerable groups, women from minority ethnic communities) not necessarily represented here.

## CONCLUSIONS

This is the first qualitative study of the IMAgiNE EURO project focusing on Italian women’s suggestions for improving QMNC during the pandemic. Participants not only experienced a lack of improvement but also a worsening of several components of QMNC during the pandemic. The latter seemed to make pre-existing issues more visible and impactful whilst also creating new challenges within health systems. Several recommendations for practice, research and education can be drawn from our findings. Women are calling for immediate action to implement postnatal care including, but not limited, to breastfeeding support, mental health care, flexibility of policies and organizational practices that can guarantee evidence-based, competent, compassionate and respectful care. This cannot be addressed without adequate implementation of staffing levels, models of care and zero-tolerance approaches reflecting national and international standards. Moreover, our findings indicate the need for an investment in human resources that goes beyond the increase of staff numbers. The greater quality of presence, communication, motivation and professionalism that women ask for should lead to reconsidering existing training and staff support within maternity services. Finally, though this study focused on the Italian context, our findings can help stakeholders and decision-makers inside and outside the Italian system, and beyond the pandemic. Further research can help to identify the most effective strategies for translating women’s suggestions for QMNC implementation into practice so to best meet the service users’ needs.

## Supplementary Material



## Data Availability

The data supporting this research are available from the authors on reasonable request.
