# Supplementary material for: Women’s suggestions on how to improve the quality of maternal and newborn care: A qualitative analysis from the IMAgiNE EURO survey in Italy during the two years of the COVID-19 pandemic
Source: Eur J Midwifery. 2024 Oct 22;8:10.18332/ejm/192143. doi: 10.18332/ejm/192143 (PMC11494818; doi:10.18332/ejm/192143)
Supplement: Supplementary file 1 [file EJM-8-62-s1.pdf]

Supplementary Table 1. Characteristics comparison between women providing or not providing a comment to the open-ended question among women who answered in Italian and who gave born in Italy.

		Women providing a comment to the open-ended question		
		Yes<br>N=2017<br>n (%)	No<br>N=5911<br>n (%)	p-value
Year of birth	2020	1311 (65.0)	3787 (64.1)	0.451
	2021	632 (31.3)	1619 (27.4)	0.001
	2022	65 (3.2)	212 (3.6)	0.442
	Missing	9 (0.4)	293 (5.0)	<0.001
Maternal country of birth	Italy	1922 (95.3)	5421 (91.7)	<0.001
	Other country	95 (4.7)	252 (4.3)	0.397
	Missing	0 (0.0)	238 (4.0)	<0.001
Maternal age range (years)	18-24	40 (2.0)	110 (1.9)	0.728
	25-30	409 (20.3)	1238 (20.9)	0.524
	31-35	878 (43.5)	2408 (40.7)	0.028
	36-39	499 (24.7)	1379 (23.3)	0.198
	≥40	191 (9.5)	540 (9.1)	0.654
	Missing	0 (0.0)	236 (4.0)	<0.001
Educational level [1]	None	0 (0.0)	3 (0.1)	0.576
	Elementary school	1 (0.0)	2 (0.0)	>0.99
	Junior High school	70 (3.5)	271 (4.6)	0.033
	High School	743 (36.8)	2276 (38.5)	0.183
	University degree	590 (29.3)	1648 (27.9)	0.237
	Postgraduate degree / Master /Doctorate or higher	611 (30.3)	1475 (25.0)	<0.001
	Missing	2 (0.1)	236 (4.0)	<0.001
Parity	1	1467 (72.7)	3829 (64.8)	<0.001
	>1	550 (27.3)	1844 (31.2)	0.001
	Missing	0 (0.0)	238 (4.0)	<0.001
Type of hospital	Public	1887 (93.6)	5217 (88.3)	<0.001
	Private	130 (6.4)	457 (7.7)	0.057
	Missing	0 (0.0)	237 (4.0)	<0.001

		Women providing a comment to the open-ended question		
		Yes	No	p-value
Mode of birth	Spontaneous vaginal birth	1250 (62.0)	3835 (64.9)	0.020
	Instrumental vaginal birth	170 (8.4)	409 (6.9)	0.028
	Caesarean section	597 (29.6)	1667 (28.2)	0.242
Infant feeding	Exclusive breastfeeding	1293 (64.1)	3930 (66.5)	0.051
	Partial breastfeeding	616 (30.5)	1635 (27.7)	0.013
	Formula	108 (5.4)	346 (5.9)	0.405
Companion allowed to stay	Always/nearly always	287 (14.2)	1332 (22.5)	<0.001
	Sometimes	451 (22.4)	1453 (24.6)	0.044
	Never/almost never	1279 (63.4)	3126 (52.9)	<0.001
Type of healthcare providers who directly assisted birth[2]	Midwife	1740 (86.3)	5017 (84.9)	0.138
	Nurse	508 (25.2)	1591 (26.9)	0.136
	A student (i.g before graduation)	104 (5.2)	284 (4.8)	0.567
	Obstetrics registrar / medical resident (under post-graduation training)	314 (15.6)	792 (13.4)	0.017
	Obstetrics and gynaecology doctor	1129 (56.0)	3205 (54.2)	0.180
	I don't know (healthcare providers did not introduce themselves)	213 (10.6)	315 (5.3)	<0.001
	Other	158 (7.8)	301 (5.1)	<0.001
Other conditions	Newborn admission in ICU/SCBU	211 (10.5)	582 (9.8)	0.452
	Maternal admission in ICU	5 (0.2)	12 (0.2)	0.922
	Multiple birth	25 (1.2)	65 (1.1)	0.696

[1]Wording on education levels agreed among partners during the Delphi. Questionnaire translated and back-translated according to ISPOR Task Force for Translation and Cultural Adaptation Principles of Good Practice; [2] More than one possible answer.

Abbreviations: ICU=intensive care unit; SCBU=special care baby unit.

© 2024 Fumagalli S. et al.
